# Multicriteria GIS-based assessment of biomass energy potentials in Nigeria

**DOI:** 10.3389/fbioe.2024.1329878

**Published:** 2024-03-19

**Authors:** M. O. Ukoba, E. O. Diemuodeke, T. A. Briggs, M. M. Ojapah, K. E. Okedu, K. Owebor, K. Akhtar, C. Ilhami

**Affiliations:** ^1^ Energy and Thermofluids Research Group, Department of Mechanical Engineering, University of Port Harcourt, Port Harcourt, Rivers, Nigeria; ^2^ Smart Energy Research Unit, Victoria University, Melbourne, VIC, Australia; ^3^ Department of Electrical and Electronics Engineering, Faculty of Engineering and Natural Science, Istinye University, Istanbul, Türkiye; ^4^ Department of Mechanical Engineering, Delta State University, Abraka, Delta, Nigeria

**Keywords:** biomass resources, residues, GIS technology, clean energy, optimal biomass plant location, carbon neutrality

## Abstract

The understanding of the geographical variability of biomass energy is an essential requirement for the optimal location of biomass energy conversion plants. This research presents a multicriteria GIS-based assessment of biomass energy potentials and the appropriate siting of biomass plants in Nigeria. The study applies the weighted overlay multicriteria decision analysis method. Crop and forest areas, settlement (energy supply areas), shrub/grasslands, barren land, water bodies, distance from water sources, road accessibility, topography, and aspect are the criteria that were considered for locating a biomass facility in this study. The results suggest that the theoretical, technical, and economical energy potentials of crop residues are highest in the North-East region of Nigeria and estimated at 1,163.32, 399.73, and 110.56 PJ/yr, respectively, and lowest in the South-East at 52.36, 17.99, and 4.98 PJ/yr, respectively. The theoretical, technical, and economical energy potentials of forest residues are highest in the North-West, estimated at 260.18, 156.11, and 43.18 PJ/yr, respectively, and lowest in the South-East at 1.79, 1.08, and 0.30 PJ/yr, respectively. Although most areas were identified to be suitable for siting biomass plants across Nigeria, the most suitable areas are located in the northern part of the country and include Niger, Zamfara, the Federal Capital Territory, Nassarawa, Kano, Kebbi, Kaduna, and Borno State. The study supports the Nigerian bio-energy policy that proposes to effectively utilize Nigeria’s non-fuelwood as a substitute for the felling of trees. This is very important to strengthen its commitment at the COP26 International Climate Conference, which is to conserve and restore its forest. Furthermore, this study will serve as a good reference for policymakers to make well-informed decisions on tackling the energy insecurity in Nigeria.

## 1 Introduction

Biomass resources from crop and forest residue have great potential and are very good sources of cleaner energy, especially in Nigeria (Ukoba et al., 2023a), where sustainable and clean energy has been a major challenge. Biomass is not only a cheap source of energy; it is readily available and considered to be carbon-neutral (Kayanta et al., 2018). Energy can be generated from biomass by utilizing biomass-to-energy conversion processes such as gasification, briquetting, biogas digestion, and direct combustion. Nevertheless, biomass application has been limited to heating, cooking, and lighting not only in Nigeria but in most developing economies (Ukoba et al., 2023b; Ogorure et al., 2018). The continuous release of atmosphere-contaminating gases such as methane (CH_4_), carbon monoxide (CO), and carbon dioxide (CO_2_) from the uncontrolled burning of biomass residues that otherwise could have served the purpose of energy generation is a great concern (Ajieh et al., 2021). Furthermore, there has been a continuous increase in energy demand that the conventional forms of energy generation cannot meet, not to mention their adverse environmental implications (Pande et al., 2021; Ben-iwo et al., 2016). Thus, it becomes important to harness renewable energy sources—biomass energy, in this case—that are not only sustainable but can also provide affordable and clean energy.

GIS and GPS data are valuable tools when assessing and analyzing biomass energy (López-Rodríguez et al., 2019; Sztubecka et al., 2020). A GIS is a very good data collection and survey tool that can work with georeferenced databases and handle volumes of data, performing arithmetic and mapping out different variables (López-Rodríguez et al., 2019). The remote sensing tool can capture Landsat imagery and other relevant data (Oyinna et al., 2023).

Studies of GIS assessment of biomass energy sources have been ongoing. Voivontas et al. (2001) developed a GIS support system to identify the distribution of biomass for electricity generation. Key parameters considered include the biomass plant facilities, locations, plant capacities, and usable spatial biomass potential distribution. Papadopoulos and Katsigiannis (2002) performed a GIS-based analysis and developed a computer program that identify optimal biomass plant site locations based on the available biomass resources and other energy-related parameters. Ranta (2005) carried out GIS-based studies on biomass potential assessment and determination of appropriate locations for biomass power plant construction. Bennui et al. (2007) conducted an integrated GIS-based multicriteria decision-making study on suitable site selection for wind turbine installation in Thailand. In the study, the analytical hierarchy program (AHP) method was utilized to weight important criteria based on their level of importance. Shi et al. (2008) employed remote sensing (RS) and GIS to evaluate feasible areas to set up new biomass plants for energy generation in Guangdong, China. The model utilized information from field surveys, statistical data from the government, and ecological and economic modeling to determine the biomass quantity and distribution. Fernandes and Costa (2010) utilized a GIS tool to assess biomass energy potential and uses of crop and forest residues in Marvão, Portugal. In the study, it was proposed that Marvão could produce approximately 10.6 ktonnes of residue annually, which is equivalent to approximately 106 TJ.

Zhang et al. (2011) carried out a GIS-based assessment and identification of the optimal location to install a forest-based biomass-to-biofuel conversion facility in Michigan’s Upper Peninsula in the United States. The research utilized a two-stage methodology in identifying the best location for siting biofuel production facilities. Jiang et al. (2012) and Cho et al. (2012) performed similar studies in the agricultural sector in China and Geoje-Hansan Bay, Korea, respectively. Kaundinya et al. (2013) and Sánchez-Lozano et al. (2014) used GIS software to identify potential areas suitable for biomass plant sites and photovoltaic solar farms. Karimzadeh et al. (2015) conducted a GIS-based multicriteria evaluation and analytic network process (ANP) algorithm for selecting landfill sites. They also employed an OWA operator in their decision-making process.

More recently, Bao et al. (2020) conducted a biomass potential assessment in Germany using a GIS tool and a dynamic yield simulation model. The assessment was conducted based on satellite data and maps of crop types, soil types, and biomass-to-bioenergy conversion factors. The research showed an increase of about 21% in transportation biodiesel/bioethanol fuel demand in 2050; however, its potential effects on irrigation and climate change were less than 3% and 4%, respectively. Pande et al. (2021) carried out a study on the development of GIS open-source applications, such as mobile, desktop, and web GIS applications, for a 10-year period and its application in environmental science (focusing on QGIS plugins). The research performed bibliometric analysis using data from VOSViewer software and Web of Science and concluded that there has been a rise in GIS applications in the last 10 years (2010–2020), especially in mobile GIS applications. Jusakulvijit et al. (2022) conducted an integrated GIS-MCA assessment with logistics analysis to ascertain bioethanol production potential from agro-residues in Thailand. The research identified suitable locations to establish a decentralized biomass plant in the region. Similar studies have been reported by La Scalia et al. (2022), Galang et al. (2022), and Ma et al. (2022) in Southern Italy, northern Cebu province in the Philippines, and China, respectively.

These published studies indicate that GIS-based analysis is capable of analyzing both spatial and non-spatial data and carrying out a multicriteria decision process. To date, optimal mapping of biomass energy facilities has not been considered in Nigeria. Yet, biomass residues are abundant in the country and could generate clean energy to support the Nigerian bio-energy policy. This research presents a multicriteria GIS-based assessment of biomass energy potentials and appropriate siting of decentralized biomass plants in Nigeria. Ten criteria were considered and include crop areas, forest areas, settlement (energy supply areas), shrub/grasslands, barren land, water bodies, distance from water source, road accessibility, topography (slope), and aspect to verify the appropriate biomass plant location, considering the spatial distribution of biomass resources. The study applies a weighted overlay multicriteria decision analysis to obtain the feasible region for siting biomass facilities to provide sustainable, affordable, and clean energy to support the UN SDGs, the Paris Agreement, and other climate mitigation pledges.

## 2 Materials and methods

A GIS tool was used to assist in the resource assessment. ArcGIS was used to conduct the GIS analysis. Remotely sensed data like the land use land cover (LULC), digital elevation model (DEM), GPS, and other primary and secondary data were gathered, integrated into the ArcGIS platform, and analyzed to produce GIS maps showing the biomass potential, and further analyzed to display suitable areas for siting biomass plants in Nigeria based on specific criteria. Ten criteria were considered in this analysis: crop areas, forest areas, settlement (energy supply areas), shrub/grasslands, barren land, water bodies, distance from water source, road accessibility, topography (slope), and aspect.

A normalized difference vegetation index (NDVI) was employed to analyze the LULC data, which include the crop areas, forest areas, settlement (energy supply areas), shrub/grasslands, barren land, and water bodies. The NDVI quantified the vegetation data that are strongly reflected (near-infrared) and those that are absorbed (RED). The estimation was done by dividing the difference between the near-infrared (NIR) and RED channels by the sum of the NIR and RED channels: $\left(NDVI=\left(NIR-RED\right)/\left(NIR+RED\right)\right)$ . This value ranges from −1 to +1; the negative values show regions covered by water, while values close to +1 indicate dense green leaves (Akbar et al., 2019).

The remotely sensed data (raster data) collected in pixel form were analyzed separately in spatial analysis and visualization due to their unique structure and format. In this study, GPS data stored in the .GPX format were imported into the GIS platform and converted into shapefile formats. In addition, the primary data with X, Y coordinates (longitude and latitude) were also imported and integrated into the GIS domain to generate a geodatabase system, which was queried and analyzed to produce better analysis and smart data-driven decisions.

Other forms of analysis, like the processing of geo data, statistical analysis, and symbolizations, were then performed on the various collected and synchronized data. The obtained results were displayed in the form of shape maps to reflect the suitable areas to locate biomass plant systems across the country.

### 2.1 Geographic location and demographic data

**FIGURE 1:**
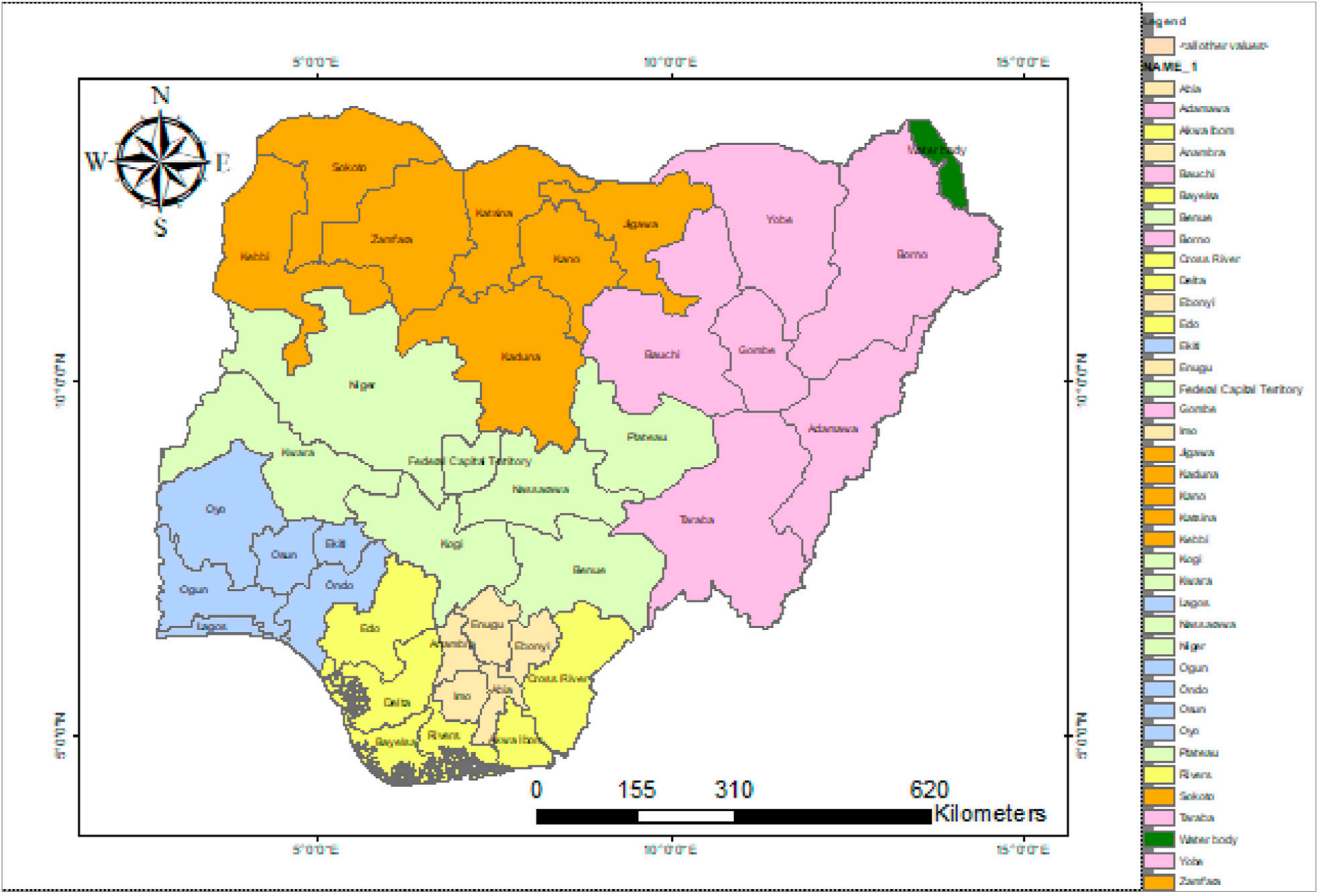
Map of Nigeria showing the various states in the country.

Nigeria is situated between longitude 2.9833 and 15.0000 [E] and latitude 3.2500 and 13.5000 [N] in the West Africa region. It shares a boundary with Chad and Cameroon to the east, Benin Republic to the west, the Gulf of Guinea to the south, and Niger to the north. It falls in the tropical region with a seasonally humid climate. Nigeria has the largest population in Africa and the seventh largest globally, with about 200 million people (Owebor et al., 2021) and a land mass of approximately 920,000 km^2^ (Ukoba et al., 2023b). Nigeria has 775 local government areas (LGAs) comprising 36 states, including the Federal Capital Territory (FCT), which are aggregated into six geopolitical zones. Figure 1 shows the map of Nigeria, including the various states and the geopolitical zones, with the North-Central (NC), North-East (NE), North-West (NW), South-East (SE), South-South (SS), and South-West (SW) symbolized with olivine yellow, rhodolite rose, electron gold, topaz sand, autunite yellow, and sugilite sky coloration, respectively.

### 2.2 Remote sensing using normalized difference vegetation index

Vegetation is quantified using NDVI by estimating strongly reflected (near-infrared, NIR) and absorbed (RED) light, considering ranges of value between −1 and +1. The possibility of water is indicated using a negative value, while the possibility of dense green leaves is indicated with values close to +1, showing a likelihood of high temperatures and tropical rainforest areas. Values from −0.28 to 0.015 indicate an area characterized by water, 0.015 to 0.14 indicate built-up areas, 0.14 to 0.18 stipulate barren lands, 0.18 to 0.27 stipulate shrub and grasslands, and 0.27 to 0.36 specify areas with sparse vegetation, while values ≥ 0.36 specify areas with dense vegetation.

#### 2.2.1 NDVI Calculation

NDVI employs NIR and RED channels to provide the characteristics of a given area. The NDVI is computed using Eq. 1.

$$NDVI=\frac{NIR-RED}{NIR+RED}.$$ (1)

### 2.3 Simulation and optimization software

A simulation tool helps determine the best location for siting biomass plants. The optimal site location may be achieved via a GIS platform, considering the stipulated weights for different criteria in order of importance.

#### 2.3.1 Criteria for site selection of the biomass plant site

Ten criteria were used to select optimal biomass plant locations. The criteria include: *crop and forest areas*: these criteria show the availability of the biomass residue feed that serves as fuel for the biomass plant; *settlement:* this criterion identifies areas where the energy generated will be supplied/utilized; *shrub/grasslands, barren land, and water bodies:* these criteria identify the grasslands, barren lands, and water bodies in the study area; *distance from road*: accessibility to the site of biomass plant facilities for transportation and maintenance; *availability of water*: water is needed for cooling and heat exchange; the *slope*: a CHP plant must be situated on a stable or flat site to mitigate sand-filling or land leveling costs at the initial stages of site preparation; the *aspect*: sunlight is required at a tolerable temperature of approximately 15°C for pretreatment and drying of the biomass residue [55–56].

#### 2.3.2 Reclassification of criteria

The LULC was classified into various categories to identify locations with high prospects. The reclassification criteria for siting biomass plants in a good location were applied on different levels based on regions with very-high, high, moderately-high, low, and very-low potential. A reclassification range of 1–10 is usually assigned, considering the potential level from lowest to highest.

#### 2.3.3 Weighted overlay analysis

The low- to high-potential regions are displayed based on a scale of 1–9 using the weighted overlay. For the crop and forest areas, the weighted overlay is done using reclassified criteria in the ArcGIS environment and then uploaded into ArcGIS before assigning a 100% weighted sum considering the influence level of each criterion. The reclassified values are matched to a scale range of 1–9 in the weighted-overlay domain.

The weighted overall score is computed using Eq. 2

$$W_{score}=\sum_{m}^{n}C_{i}*W_{i},$$ (2)

$$i=\left\{LULC\;resource,\text{distance from road},\text{distance from water},\text{slope},\text{aspect}\right\},$$

where $W_{score}$ is the overall weighted overlay score, $C_{i}$ is the criterion score of i, and $W_{i}$ is the weight value of criterion i .

#### 2.3.4 Suitability analysis

The weighted overlay obtained result is further analyzed using the Map Analyst tool in the raster calculator section in ArcGIS to get the most suitable area for siting the plant. The suitability area (SA) calculation based on the criteria is performed using Eq. 3:

$${SA}_{i}=W_{i}\times LULC\;;\;i=\left(crop,forest\right),$$ (3)

where ${SA}_{i}$ represents the suitability area, $W_{i}$ represents weighted vegetation, and LULC represents the land use and land cover.

**FIGURE 2:**
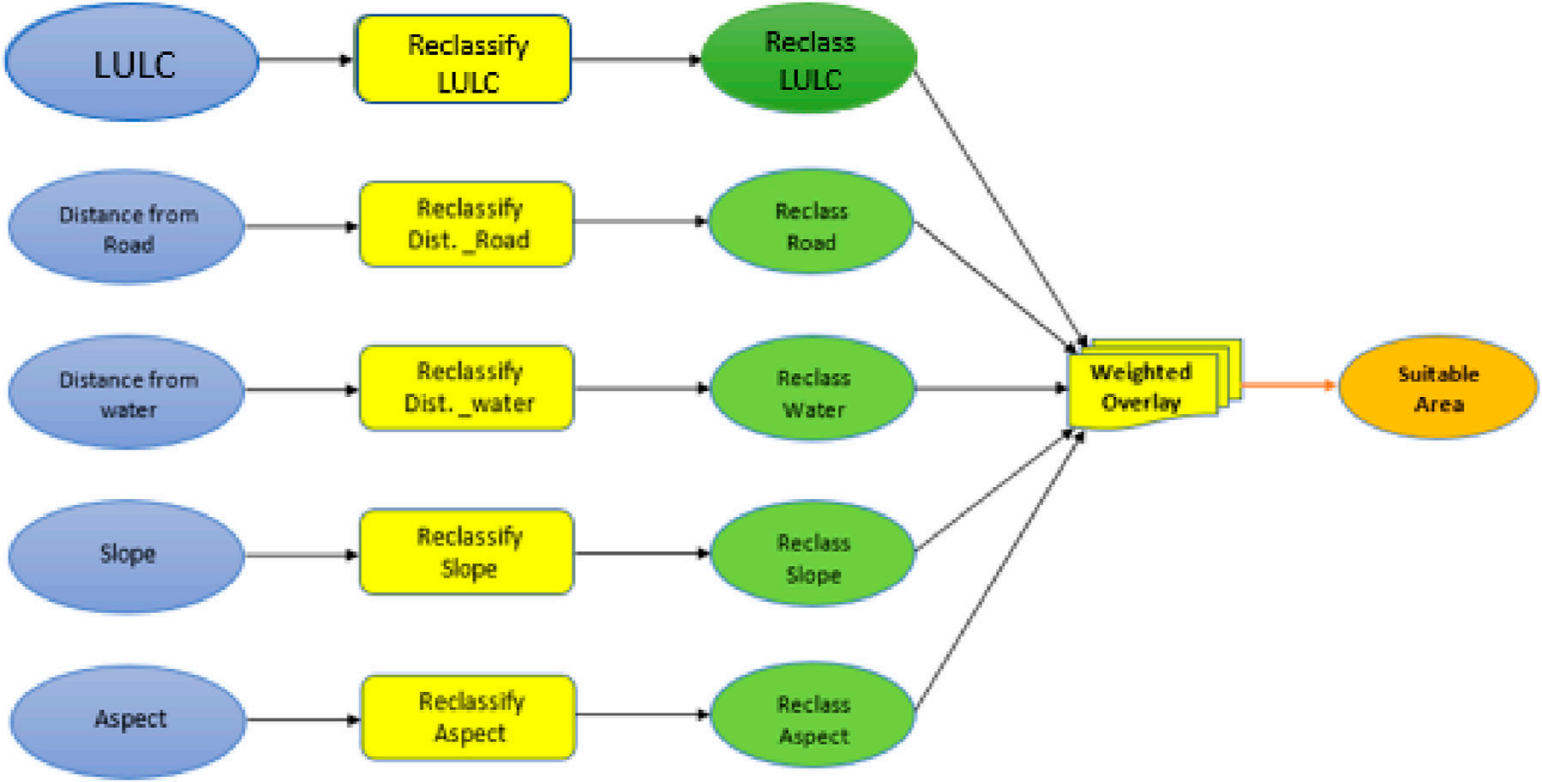
Suitability analysis model.

Figure 2 shows the suitability analysis model used to identify the suitable areas for siting the biomass plant.

### 2.4 Crop/forest residue

#### 2.4.1 Theoretical assessment

The theoretical assessment considers the peak available amount of biomass resources to generate energy apart from the amount required for food or industrial purposes (Hassan et al., 2019), considering the specific region, cultivation area, and the net biomass yield (obtained based on variables such as conditions of the climate, soil, and biomass features). The annual biomass energy that is available from crop and forest residues (Ukoba et al., 2023b; Souza et al., 2021) is also identified. The following are the main properties of biomass: production rate, low heating value (LHV), residue-to-product ratio (RPR), and estimated residue, which is the product of the crop production rate and the mean RPR. From Eq. 3, the product of the residue potential and the effective mean energy content of the residue is taken to obtain the weighted overlay. Consequently, the energy potential (Eq. 4), in theory, is (Ben-iwo et al., 2016)

$$E_{theoretical}=\sum_{i}^{n}F_{j}*LHV;\;j=\left\{crop,forest\right\},$$ (4)

where $E_{theoretical}$ is the theoretical energy potential, LHV is the low heating value or mean energy content ( ${\bar{E}}_{c}$ )[kJ/kg], and $F_{j}$ is the residue potential or obtainable residue [ktonnes] given according to Eq. 5.

$$F_{crop}=\sum_{i}^{n}\;P*\bar{RPR},$$ (5)

where P is crop production [ktonnes], and $\bar{RPR}$ is the mean residue-to-product ratio [-].

The mass forest product volume (m^3^) is expressed as Eq. 6 for the forest residue.

$$m_{F}=\rho \times V,$$ (6)

where $m_{F}$ is the mass of the forest product, ρ is the density of the forest product, and V is the volume of the forest product.

The forest residue can be obtained from Eq. 7:

$$F_{forest}=m_{F}\times RPR,$$ (7)

where $F_{forest}$ is the forest residue and RPR is the residue-to-product ratio, which can be assumed to be 0.72 (Ukoba et al., 2023b).

The estimated energy content or LHV of wood fuel and wood charcoal can be assumed to be 19.5 MJ/kg and 28.0 MJ/kg, respectively, according to Bhattacharya et al. (2002).

#### 2.4.2 Technical assessment

The fraction of the theoretical energy potential that could be effectively utilized for energy purposes is known as the technical assessment. The technical potential depends on the theoretical residue potential on an annual basis. Thus, an availability factor (A_F_) is considered to indicate the amount of the residue that can be utilised for energy generation yearly. The range of A_F_ is 01–1 and changes due to location and the crop residue, as reported in Ukoba et al. (2023b) and Souza et al. (2021). The technical potential is computed according to Eq. 8

$$E_{technical}=\sum_{i}^{n}E_{theoretical}*A_{F},$$ (8)

where $E_{technical}$ is the technical potential and $A_{F}$ is an availability factor that ranges from 0 to 1.

Availability factors ( $A_{F}$ ) of 0.4, 0.5–0.75, and 0.8 were assumed for rice residue, wood residue, and oil-palm residues, respectively (Ukoba et al., 2023b; Souza et al., 2021; Portugal-Pereira et al., 2015) while 0.30 was used for the other crops because all agro-crops share a similar availability factor $\left(A_{F}\right)$ range according to the reports of Ukoba et al. (2023b) and Deng et al. (2015). Moreover, all the forest residues in Nigeria were assigned an $A_{F}$ of 0.6, in line with Ukoba et al. (2023b).

#### 2.4.3 Economic assessment

The portion of the technical potential that determines the economic profitability criteria in a certain condition (Thorenz et al., 2018) is known as the economic assessment. According to Gómez et al. (2010), the collection area of the biomass residue plays a huge role in the total cost of generating electricity. With a large area of residue collection, there is more room for installation of a high-capacity biomass plant that is more cost beneficial. The cost of transporting the residue to the site falls under operational costs and contributes a significant part of the total generation cost of power (Noon and Daly, 1996). Therefore, some constraints on the viability of the biomass residue are factors such as biomass residue collection, processing, and transportation.

It should be noted that not all the available biomass residues contain useful energy. Consequently, it is imperative to know the optimal economic transportation radius (Souza et al., 2021). GIS has been proven to be a proficient tool for determining optimal distances for the transportation of biomass residue (Souza et al., 2021; Haase et al., 2016). The optimal feasible distance is not constant as it changes based on the location with a range from 30 km to 100 km (Portugal-Pereira et al., 2015) with an economic radius of 24%–59% of the technical potential (Gómez et al., 2010; Haase et al., 2016; Lopez et al., 2012). Eq. 9 is used to evaluate the economic potential as follows:

$$E_{economic}=\sum_{i}^{n}E_{technical}*r,$$ (9)

where $E_{economic}$ is the economic potential, and r is the economic radius [%].

This article uses the feasible distance or economic radius as 27.66% of the collection area for the initial approximation; this value is in line with the Souza et al. (Ben-iwo et al., 2021) study in Brazil.

## 3 Result and discussion

### 3.1 Land cover classification and analysis

**FIGURE 3:**
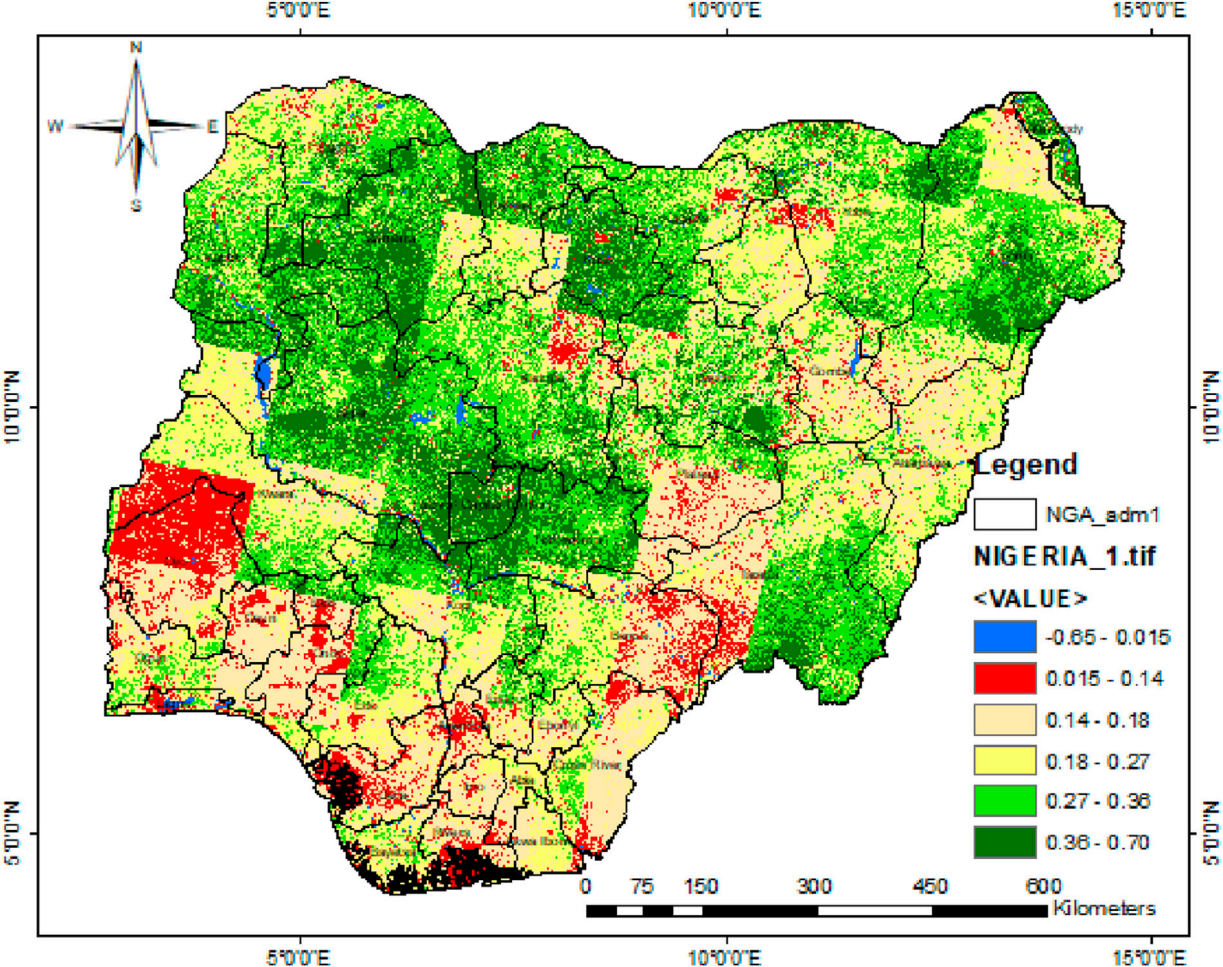
Nigerian biomass distribution.

Based on the described methodology, the Nigerian NDVI classification ranges for six LULCs derived from Landsat-8 OLI data (USGS, 2021) are displayed in Table 1. The classification range corresponds with Akbar et al.’s (2019) research, aside from the initial (water body) and final (dense vegetation) classification range, which varies based on the geographical location. The LULC map of Nigeria based on the NDVI classification ranges is presented in Figure 3.

**TABLE 1:** NDVI classification range for land cover in Nigeria.

Classification	Label	NDVI range	Colors
1	Water body	−0.65–0.015	Cretan blue
2	Built-up area	0.015–0.14	Mars red
3	Barren land	0.14–0.18	Topaz sand
4	Shrub and grassland	0.18–0.27	Autunite yellow
5	Sparse vegetation (crop area)	0.27–0.36	Light (quetzel) green
6	Dense vegetation (forest area)	0.36–0.70	Dark (fir) green

**FIGURE 4:**
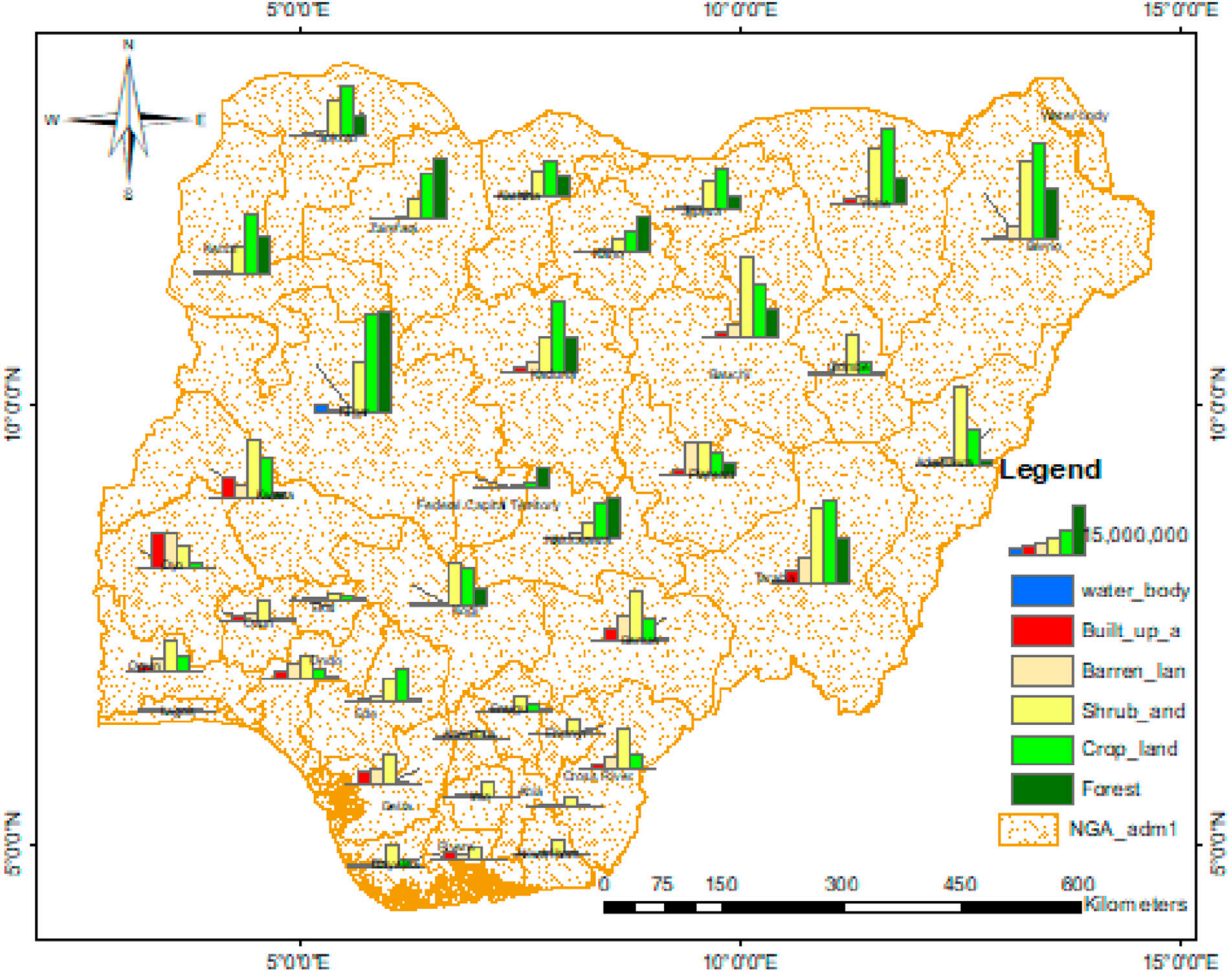
GIS bar chart map of Nigeria’s LULC count.

**FIGURE 5:**
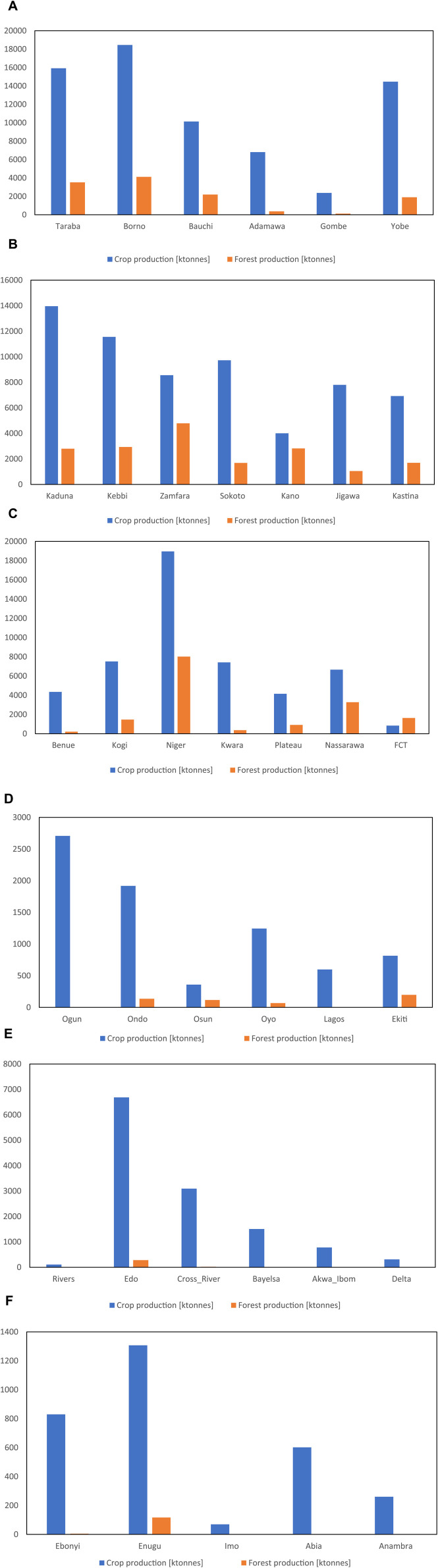
(Continued).

The crop and forest area counts were captured from the Landsat-8 data obtained from USGS (2021) (See Figure 4). They were analyzed using the country’s total crop production in 2019 and total forest production in 2020 (FAO, 2021) based on the state counts to get the estimated crop and forest production across the various states in Nigeria, as presented in Figures 5A–F.

Figure 5A shows the crop and forest production by state in the North-East zone, where Borno State is the highest crop and forest-producing state (18,456,427.31 and 4,123,891.16 tonnes per year), followed by Taraba (15,923,044.46 and 3,522,180.17 tonnes per year) and then Yobe (14,462,983.83 and 1,904,296.22 tonnes per year).

Figure 5B shows the crop and forest production by state in the North-West zone where Kaduna State is the highest crop-producing state (13,960,218.20 tonnes per year), followed by Kebbi (11,558,167.74 tonnes per year) and then Sokoto (9,718,616.05 tonnes per year). For forest production, Zamfara is the highest-producing state (4,787,048.96 tonnes per year), followed by Kebbi (2,934,235 tonnes per year) and then Kano (2,818,670.10 tonnes per year).

For the North-Central zone shown in Figure 5C, Niger State is the highest crop- and forest-producing state (18,955,373.18 and 8,018,194.86 tonnes per year, respectively), followed by Kogi (7,511,411.46 tonnes per year) and then Kwara (7,413,940.86 tonnes per year) for crop production. For forest production, Nasarawa followed Niger State with 3,275,121.58 tonnes per year, followed by FCT, which was grouped under NC (1,464,467.15 tonnes per year) and then Kogi (1,464,467.14 tonnes per year).

Figure 5D shows the crop and forest production by state in the South-West zone where Ogun State is the highest crop-producing state (2,707,388.75 tonnes per year), followed by Ondo (1,918,582.63 tonnes per year) and then Oyo (1,244,261 tonnes per year). For forest production, Ekiti is the highest (197,750.28 tonnes per year), followed by Ondo (135,888.93 tonnes per year) and then Osun (116,148.66 tonnes per year). For the South-South zone, as shown in Figure 5E, Edo State is the highest crop- and forest-producing state (6,684,605.97 and 281,198.92 tonnes per year, respectively), followed by Cross-River (3,095,118.16 and 17,048.98 tonnes per year, respectively) and then Bayelsa (1,505,658.23 and 1,349.57 tonnes per year, respectively).

For the South-East zone shown in Figure 5F, Enugu State is the highest crop- and forest-producing state (1,307,251.95 and 116,540.19 tonnes per year, respectively), followed by Ebonyi (829,647.60 and 4,689.22 tonnes per year, respectively) and then Abia (601,723.10 tonnes per year) for crop production. For forest production, Anambra followed Enugu and Ebonyi State with 793.78 tonnes per year.

**FIGURE 6:**
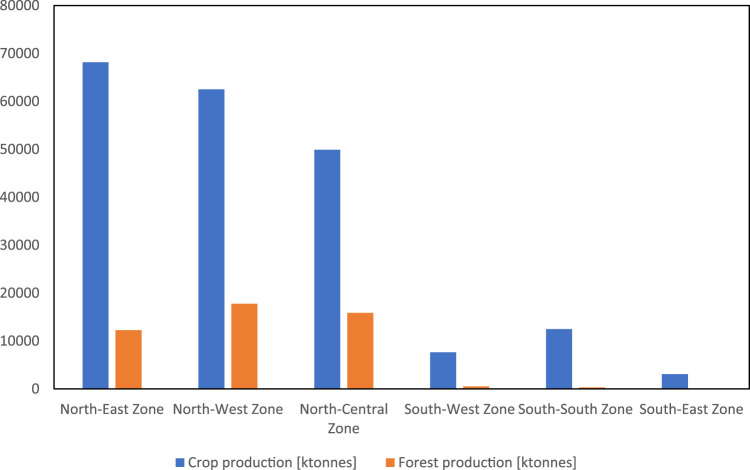
Nigerian crop and forest production by zones [tonnes].

Figure 6 shows that northern Nigeria is the highest region for crop and forest production across Nigeria. Crop production by zone is led by NE, followed by the NW, NC, SS, SW, and SE. Forest production is led by NW, followed by NC, NE, SW, SS, and SE.

### 3.2 Suitability analysis using weighted overlay in ArcGIS

#### 3.2.1 Reclassification of criteria

Various criteria are considered to identify the optimal areas for siting biomass plants in Nigeria: crop, forest, settlement, shrub/grasslands, barren land, and water body areas (embedded in LULC), distance from a water source and road accessibility (GPS data), and slope and aspect (DEM data). These criteria were classified into various categories (See Table 2). The reclassification was done to make all the parameters dimensionless for easy query and analysis.

**TABLE 2:** Weight, influence, sub-criteria, and ranks using weighted overlay in ArcGIS.

S/N	Criteria	Assigned weight	Influence (%)	Sub-criteria	Reclass value	Weighted rank
A	LULC features	0.50	50			
1	Water			−0.65–0.015	1	2
2	Settlement (built-up areas)			0.015–0.14	2	7
3	Barren land			0.14–0.18	3	3
4	Shrubs and grassland			0.18–0.27	4	5
5	Crop land			0.27–0.36	5	9
6	Forest land			0.36–0.70	6	9
7	Distance from road [km]	0.20	20	0.5–0.501	9	9
				0.501–1	8	8
				1–1.5	7	7
				1.5–2.0	5	5
				2.0–2.5	3	3
				2.5–3.0	2	2
				>3.0	1	1
8	Distance from river [km]	0.15	15	0.5–0.501	9	9
				0.501–1	8	8
				1–1.5	7	7
				1.5–2.0	5	5
				2.0–2.5	3	3
				2.5–3.0	2	2
				>3.0	1	1
9	Slope	0.08	8	0–5	10	9
				5–10	9	8
				10–15	8	7
				15–20	7	6
				20–25	6	5
				25–30	5	4
				30–35	4	3
				35–40	3	2
				40–45	2	1
				>45	1	1
10	Aspect	0.07	7	−1–0 (Flat)	10	9
				0–22.5 (N)	1	1
				22.5–67.5 (NE)	3	3
				67.5–112.5 (E)	5	5
				112.5–157.5 (SE)	9	8
				157.5–202.5 (S)	9	8
				202.5–247.5 (SW)	10	9
				247.5–292.5 (W)	5	5
				292.5–337.5 (NW)	3	3
				337.5–360.0 (N)	1	1

**FIGURE 7:**
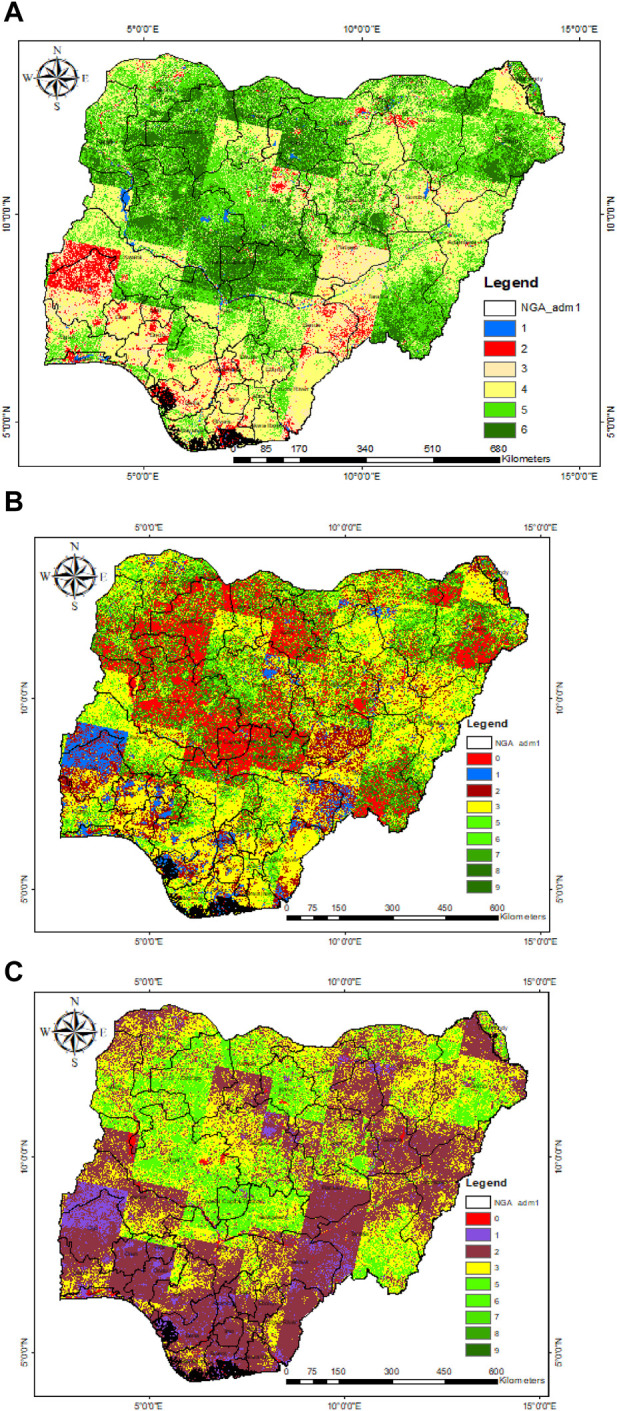
**(A)** Reclassified LULC. **(B)** Reclassified crop lands. **(C)** Reclassified forest lands.

Figure 7A shows the reclassification of the LULC criteria used for the suitability analysis in the ArcGIS platform. The classification was done in five (5) levels. The dark green, light green, yellow, red, and blue colors depict regions with very-high, high, moderately high, low, and very-low potential, respectively. Based on the potential level of the criteria, a classification range of 1–10 is assigned to indicate the potential level from the lowest to the highest.

Figures 7B, C show the reclassification of crop and forest lands. Figure 7B indicates areas suitable for biomass plant siting, considering crop residue as feedstock for the biomass-plant system based on criteria including crop, settlement, shrub/grasslands, barren land, and water bodies. Figure 7C shows suitable areas considering forest residue as feedstock for the biomass-plant system based on criteria including forest, settlement, shrub/grasslands, barren land, and water bodies.

#### 3.2.2 Weighted overlay analysis

The reclassified parameters were uploaded into the weighted overlay platform in ArcGIS and assigned a weighted percentage based on their weighting influence (level of importance), as shown in Table 2. Table 2 also shows the sub-criteria and ranks. A scale of 1–9 was utilized to indicate low–high potential regions, respectively, in the weighted overlay analysis displays.

#### 3.2.3 Suitability analysis

Further analysis was performed on the weighted overlay result using the raster calculator in the Map Analyst domain in ArcGIS to obtain the optimal regions for biomass-to-energy plant siting.

**FIGURE 8:**
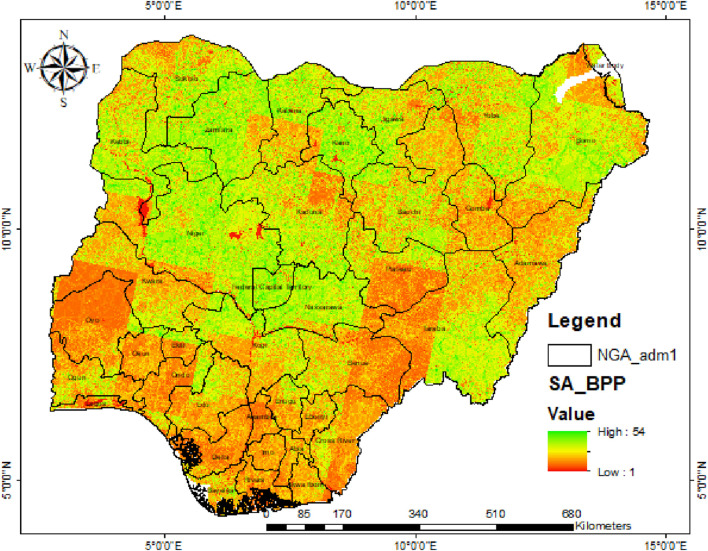
Suitable areas for siting biomass power plants in Nigeria.

Figure 8 presents the most suitable areas (SAs) for biomass plant siting, considering 10 criteria, including crop, forest, settlement, shrub/grasslands, barren land, water bodies, distance from water sources, road accessibility, topography, and aspect.

The analysis performed is similar to that Ukoba et al. (2023a) carried out for Edo State, Nigeria. The analysis indicates that the theoretical, technical, and economical energy potentials of crop residues are highest in the North-East region of Nigeria and are estimated at 1,163.32, 399.73, and 110.56 PJ/yr, respectively, and lowest in the South-East at 52.36, 17.99, and 4.98 PJ/yr, respectively. The theoretical, technical, and economical energy potentials of forest residues are highest in the North-West, estimated at 260.18, 156.11, and 43.18 PJ/yr, respectively, and lowest in the South-East at 1.79, 1.08, and 0.30 PJ/yr, respectively. Although most areas were identified to be suitable for siting biomass plants across Nigeria, the most suitable areas are located in the northern part of the country and include Niger, Zamfara, the Federal Capital Territory, Nassarawa, Kano, Kebbi, Kaduna, and Borno State.

## 4 Conclusion

Biomass residues are attractive energy generation feedstock. Meanwhile, biomass resource estimation is a challenging task, especially where there is little to no data. Utilizing a remote sensing application alongside GIS techniques can provide high-resolution mapping of the biomass resource distribution across any region of interest. This article presented a multicriteria GIS-based assessment of biomass energy potentials and appropriate siting of biomass plants in Nigeria. The study serves as a good reference/guide for policymakers to make well-informed decisions on tackling the energy insecurity in Nigeria. It applies the weighted overlay multicriteria decision analysis with 10 criteria that include crop areas, forest areas, settlement (energy supply areas), shrub/grasslands, barren land, water bodies, distance from water source, road accessibility, topography (slope), and aspect to find the best locations for siting biomass facilities in Nigeria. ArcGIS was used to conduct the GIS analysis, while RS and other primary/secondary data were collected and integrated into the ArcGIS platform to form a geodatabase system, which was queried and analyzed to create reliable and smart data-driven decisions. Key findings reveal that the northern zones (North-East, North-West, and North-Central) are the highest crop and forest production zones in Nigeria, and thus, they have the highest residue generation in the country.

From the findings, the estimated crop residue theoretical, technical, and economic energy potential is highest in the North-East of Nigeria (1,163.32, 399.73, and 110.56 PJ/yr, respectively), followed by the North-West (1,066.76, 366.55, and 101.39 PJ/yr, respectively), North-Central (851.16, 292.47, and 80.90 PJ/yr, respectively), South-South (213.01, 73.19, and 20.25 PJ/yr, respectively), South-West (130.42, 44.81, and 12.40 PJ/yr, respectively), and lowest in the South-East (52.36, 17.99, and 4.98 PJ/yr, respectively). The estimated theoretical, technical, and economic energy potential of forest residues are highest in the North-West of Nigeria (260.18, 156.11, and 43.18 PJ/yr, respectively), followed by the North-Central (232.54, 139.53, and 38.59 PJ/yr, respectively), the North-East (179.32, 107.59, and 29.76 PJ/yr, respectively), the South-West (7.68, 4.61, and 1.27 PJ/yr, respectively), the South-South (4.39, 2.64, and 0.73 PJ/yr, respectively), and lowest in the South-East (1.79, 1.08, and 0.30 PJ/yr, respectively). Although there are suitable areas for siting biomass plants across the various states in Nigeria, the most promising sites are in Niger, Zamfara, FCT, Nassarawa, Kano, Kebbi, Kaduna, and Borno State, all located in the northern regions of Nigeria.

## 5 Limitations

The present work is limited to the assessment of biomass energy potentials and appropriate siting of biomass plants in Nigeria. It suggests possible biomass conversion technologies but does not consider the analysis of such biomass conversion plants for energy generation in the most suitable location. Furthermore, the research focused on geography and environment as the criteria for choosing an optimal location. Thus, it is limited in that it did not consider socioeconomic factors as part of the decision criteria. Further studies could consider environmental, geographical, and socioeconomic factors in determining the optimal site.

## Data Availability

The original contributions presented in the study are included in the article/supplementary material; further inquiries can be directed to the corresponding author.
